# The Yin and Yang of Sodium Lauryl Sulfate Use for Oral and Periodontal Health: A Literature Review

**DOI:** 10.30476/dentjods.2022.95108.1836

**Published:** 2023-09

**Authors:** Hamoun Sabri, Mohammad Moein Derakhshan Barjoei, Ali Azarm, Negar Sadighnia, Reza Shakiba, Ghazal Aghebati, Negin Hadilou, Parisa Kheiri, Fariba Ghanbari, Niloofar Deravi, Melika Mokhtari

**Affiliations:** 1 Dept. of Periodontics and Oral Medicine, University of Michigan Dental School, Ann arbor, MI, USA; 2 Student Research Committee, Shahid Sadoughi University of Medical Sciences, Yazd, Iran; 3 USERN Office, Shahid Sadoughi University of Medical Sciences, Yazd, Iran; 4 Student Research Committee, Rafsanjan University of Medical Sciences, Rafsanjan, Iran; 5 Student Research Committee, Tabriz University of Medical Sciences, Tabriz, Iran; 6 School of Dentistry, Mashhad University of Medical Sciences, Mashhad, Iran; 7 Dept. of Pediatric Dentistry, Dental School, Mashhad University of Medical Sciences, Mashhad, Iran; 8 Student Research Committee, School of Medicine, Shahid Beheshti University of Medical Sciences, Tehran, Iran; 9 Student Research Committee, Dental Faculty, Tehran Medical Sciences, Islamic Azad University, Tehran, Iran

**Keywords:** Sodium Lauryl Sulfate, Oral medicine, Drug effects, Oral mucosa, Oral cavity, Periodontal health

## Abstract

Sodium lauryl sulfate (SLS) is an anionic surfactant, which has a wide range of usage in the health sector and in dental pharmaceutical products, especially in toothpastes. The objective of this review was to investigate the effects of SLS containing dentifrices on oral and periodontal health, possible side effects, and its benefits. A thorough literature search was done using databases of PubMed and Google Scholar and finally, 40 articles were included in the study. This narrative review revealed the sources of discrepancy and conflicting results regarding the impact of SLS on oral cavity as well as a lack of sufficient evidence in most topics. Hence, the evidence suggests improved drug bioavailability when used as a solubilizer, improved plaque control, and reduction in bad breath. On the other hand, SLS can serve as a risk indicator of prolonged oral wound healing time, recurrent aphthous stomatitis.

## Introduction

Sodium lauryl sulfate (SLS) with the chemical formula of “C12H25NaO4S” is an anionic surfactant, in other words, it is the sodium salt of lauryl alcohol (1-Dodecanol) and, it is structured as sulfuric acid mono-dodecyl ester sodium salt [ 1 ]. Its usual concentration varies from 0.5 to 2%, which is used as a detergent in the house cleaning and dishwashing products and soaps [ 1 ]. In addition, SLS has a wide range of usage in the health sector and in pharmaceutical products such as cosmetic products, shampoos, hand soaps, and so on [ 1 - 2 ].

The application of SLS in the field of dentistry goes back to more than 50 years ago, and it has been commonly used in dentifrices like toothpastes [ 2 - 4 ]. Regarding its solubilizing potential, it is widely used in solid oral dosage formulation to increase the solubility of poorly dissoluble drugs [ 2 - 6 ]. Not only is it a wetting agent in oral health products, but also it increases the solubility of lipids and flavors. It has a direct antimicrobial effect owing to its adsorption and penetration through the porous cell wall followed by interaction with the components of the cell membrane lipids and proteins [ 1 , 5 ]. Furthermore, it maximizes the foaming action and reduces the surface tension of water, which allows a better application of toothpastes [ 5 ]. 

However, adverse effects of SLS also have been reported [ 7 ]. Rubright *et al*. [ 7 ] were one of the pioneers who reported the side effects of SLS in oral health. These effects mostly consist of dose-dependent irritative dermal reactions in high-dose usage as well as oral mucosa desquamation and reduction in the function of the protective barrier of oral epithelium due to multi factors [ 7 ]. Moreover, oral epithelium shedding, swelling, and ulceration have also been observed [ 5 , 8 ]. In one study in animal models, Ahlfors and Lyberg [ 8 ] reported that sensitivity to low concentrations of SLS is much higher for the oral mucosa than the skin. Whereas other reports showed that SLS usage dries up the oral mucosal protective layer and exposes the buccal mucosa and gingiva to irritants [ 9 - 11 ]. However, SLS may also denature the proteins of mucosa considering its affinity to them [ 5 ].

As mentioned in the previous paragraph, the consumption of SLS-containing products may lead to various phenomena. However, it is still unclear whether the administration SLS-containing dentifrices would directly affect the oral cavity and alter the mucosal condition. Therefore, the purpose of the present review is to investigate the effects of SLS-containing dentifrices on periodontal and oral health, and evaluate its possible side effects or benefits.

## Search Strategy

A meticulous search was conducted using PubMed and Google Scholar databases. A limitation of 21 years (2000-2021) was applied. The references list of all selected articles were also hand-searched by one of the authors to detect additional potentially relevant studies. The search query for Medline (PubMed) was ((“sodium lauryl sulfate”) ) AND ((treatment) OR (effect) OR (influence) OR (usage)) AND ((mouth) OR (stoma) OR (dental) OR (oral) OR (periodontal)): from 2000 – 2021 and the same strategy with the keywords of “Sodium lauryl sulfate” AND “effects” AND “oral”, ” mouth” , “periodontal” in the Google scholar was performed. One author (H.S) conducted the narrative review search, and the articles were selected for full-text reading independently by two authors based on titles and abstracts.

### Inclusion and exclusion criteria

Published articles in both English and non-English languages were considered if they contained any detailed data about the SLS exposure in oral mucosa. Moreover, the non-English articles were included, should they contain conclusive data within their English abstracts. Both human and animal studies were included. Articles, which included data about SLS exposure on human skin or other mucosal membranes rather than oral mucosa, were excluded. 

## Results

**Figure 1:**
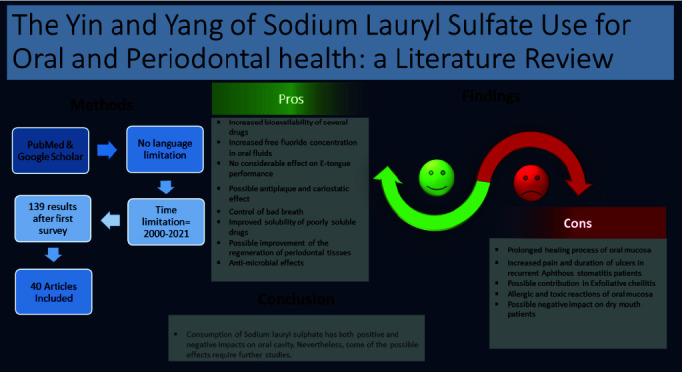
Graphical summary of the search process and the findings

After removing duplicates, two authors independently performed title, abstract, and full-text screening for the articles that could not be screened properly by title and abstract. Out of 189 articles found by the search strategy, 49 were selected for the primary evaluation. After the primary evaluation by the same two authors and considering the inclusion and exclusion criteria, 40 articles were included in the study. Table 1 illustrates a brief outcome of each included article. Based on our findings from the search protocol, the included articles were categorized according to the most suitable topic. Figure 1 depicts the reviewing process and the summary of the results.

**Table 1:** Study characteristics of included studies

Category	Type of study	*In vitro*/ *In vivo*	SLS exposure	Outcome	References
Effects on free fluoride concentration in oral fluids	RCT	*In vivo* (human)	48 h massed plaque, before washing with a 12 mmole/l NaF (228 μg/g F rinse) mouthwash with 0.5% SLS or without 0.5% SLS	SLS had small effect on total plaque fluoride. SLS made a small non-significant increase in total saliva fluid. SLS significantly increased plaque fluid and salivary fluid fluoride	[ 1 ]
Wound Healing	Experimental	*In vitro*	HGFs cultures took one of the SLS order: from 0.00% (control), to 0.05% SLS (w/v) (with 0.01 interval between group) in media containing 5% FBS, for 2 minutes. Cultures termination on days 0, 2, 4, 6 and 8	SLS significantly inhibited wound healing	[ 2 ]
Impact on e-tongue device	Experimental	*In vitro*	Solution with 1% SLS tested on electronic tongue.	SLS changes the “test” signal sensor sets in compared to control sensor.	[ 3 ]
				The performance of the sensor was not harmed by this change	
Management of halitosis	Experimental	*In vivo*	0.005-5% SLS + cell-free FTF enzyme and fructans	FTF activity and ECPs structure changes decreased	[ 4 ]
Management of halitosis	RCT	*In vivo* (human)	SLS (0 %, 1.1 %, 2.2%) in detergent	Sulfide gas decreased significantly ammonia decreased but not significantly	[ 5 ]
Plaque index	RCT	*In vivo* (human)	toothpastes (0%, 1.1% and 2.2% SLS) for 4 weeks.	increased SLS concentration is associated with decreased plaque control and Salivary flow but not significantly	[ 6 ]
Cytotoxicity	Experimental	*In vitro*	2% SLS + cementum for 1, 3 and 5 minutes.	SLS can remove the root surface completely and partially dependent to exposure of time.	[ 7 ]
Solubilizer	RCT	*In vivo* (human)	1-5% SLS and non-SLS toothpaste for 8 weeks	SLS and non-SLS toothpastes showed same efficacy nevertheless containing one seems more pleasant for patients	[ 8 ]
Effects on saliva	RCT	*In vivo* (human)	1% SLS only, 4% betaine only, 1% SLS- 4% betaine containing and control toothpastes for 6 weeks	Other ingredients of toothpastes might be more responsible for mucosal irritating effects rather than SLS	[ 9 ]
EC	Case-report	*In vivo* (human)	SLS containing toothpaste	SLS might be a responsible element EC	[ 10 ]
Recurrent aphthous stomatitis	Crossover RCT	*In vivo* (human)	Usual brushing method + dentifrice and toothbrush supplied. Three dentifrices	SLS-containing toothpastes affected the ulcer healing process and it was significantly lower in SLS-free group.	[ 11 ]
			1. A commercially available SLS-free dentifrice		
			2. A dentifrice containing 1.5% SLS	Patients from these group reported more pain in daily lives	
			3. A commercially available 1.5% SLS-containing dentifrice		
Recurrent aphthous stomatitis	Systematic review	*In vivo* (human)	4 crossover clinical trials: systematic review meta-analysis: 2 clinical trials	SLS‐free dentifrice significantly reduced the ulcers’ number, ulcer duration, episodes’ number, and ulcer pain compared to SLS‐containing	[ 12 ]
Carrier for various oral drugs	RCT	*In vivo* (rat)	Dissolved in water, 2% solution	Significant only in ileum	[ 13 ]
Carrier for various oral drugs	Experimental	*In vitro*	0.5% w/v SLS in water	The CMC of SLS:	[ 14 ]
				water> FeSSIF> SGF	
				aggregation of SLS:	
				SGF>FeSSIF>water	
				Optimum solubility happened when 2 mg of SLS was used.	
Carrier for various oral drugs	Experimental	*In vitro*	Anionic form SLS Water based solution	SLS has no - effect on e-tongue sensors	[ 3 ]
Carrier for various oral drugs	Experimental	*In vitro*	2:1 SLS : mirabegron Salt	SLS reduced solubility of the drug and slows down drug release, for it has sulfate and alkyl groups	[ 15 ]
Carrier for various oral drugs	Experimental	*In vitro*	Dried form and Suspension form of SLS salt/complex and microparticles containing SLS salt/complex	The microparticles have slower dissolution profiles than LS salt/ complex. There were no significant differences between dissolution profiles of suspensions and dried forms of salt/complex and microparticles containing LS salt/complex	[ 16 ]
Carrier for various oral drugs	RCT	*In vivo* (rats)	3 groups Mirabegron alone as solution (1.25mg/mL), SLS/drug suspension, SLS/drug microparticles suspension	The microparticle suspension showed a better performance in dogs than LS salt/complex suspension. In mirabegron alone group, maximum concentration of the drug in plasma was higher in the fasting group that could get rapidly toxic. Using a suspension, the difference between fasting and fed groups was decreased.	[ 16 ]
				Microparticle suspension produced similar results under fasted and fed conditions.	
Carrier for various oral drugs	Experimental	*In vitro*	19 drugs (Acetaminophen, Benzoic Acid, Budesonide, Carbamazepine, Carvedilol, Celecoxib, Enrofloxacin, Glibenclamide, Ibuprofen, Indomethacin, Ketoconazole, Lamotrigine, Mycophenolate, mofetil, Phenothiazine, Naproxen, Phenytoin, Piroxicam, Salicylic Acid, Tadalafil)+SLS (0.5% & 0.1%)	The solubility of most drugs increased (different among drugs, Acetaminophen the least & Ketoconazole the most)	[ 17 ]
Carrier for various oral drugs	Experimental	*In vitro*	150 mg BILR355+ SLS & PVP (1:1 w/w), SLS +excess API in 7 mL water + 0.01% to 1.0% (w/v) or (0.35 to 34.7 mM)	SLS spectrum > Cognis for BILR 355 dissolution but both were good.	[ 8 ]
Carrier for various oral drugs	Experimental	*In vitro*	pre-dissolved HPMC-AS or SLS (0.3, 1, or 3 mg/mL) + (1 & 3 mg/ml HMPC-AS), LLPS (amorphous precipitates)	SLS increased PSZ solubility+ synergism with HMPC, SLS (3 mg/ml) reduced the precipitation of PSZ & crystallization inhibition not useful for *in vivo* LLPS increased drug bioavailability	[ 18 ]
Carrier for various oral drugs	Experimental	*In vivo* (rats)	A nanosuspension for Isradipine containing: SLS + vitamin E + TPGS (particle size = 539 nm)	The particle size reduction can influence ISR absorption in gastrointestinal tract and thus nanosuspension technology is responsible for the increase of oral bioavailability in rats.	[ 19 ]
Carrier for various oral drugs	Experimental	*In vitro*	SLS as an oral mucosal penetration enhancer for Pravastatin Sodium tablets	Muco-adhesive layered buccal tablets containing 1% SLS produced a good mucoadhesive strength, 96% drug release over 2 h, and 23% permeation of the drug through buccal mucosa without any tissue damage.	[ 20 ]
Carrier for various oral drugs	RCT	*In vivo* (human)	Accumulated plaque for 48 h before rinsing with a 12 mmole/l NaF (228 μg/g F) rinse containing or not containing 0.5% (w/w) SLS	SLS had no statistically significant effect on total plaque and total saliva fluoride but significantly increased salivary fluid and plaque fluid fluoride.	[ 1 ]
Cytotoxicity	experimental	*In vitro*	Toothpaste and mouthwash	SLS should be replaced with safer detergents	[ 21 ]
Cytotoxicity	experimental	*In vivo* (rabbit, rat)	Gel SLS (2%, w/w)vaginal, Rectal and Penile mucosaEye, Skin, Buccal mucosa	gel formulation containing the 2%ww of SLS, can be considered safe for the buccal mucosa.	[ 22 ]
Enamel erosion	experimental	*In vitro*	SLS Solution with concentrations of 1.0 and 1.5%	The protection of fluoride decreased in the initial erosion, but this effect did not remain with the preservation of the erosive cycle.	[ 19 ]
Mucosal reactions	Case-report	*In vivo* (human)	Toothpaste containing SLS	oral lesions	[ 23 ]
Mucosal reactions	Case-report	*In vivo* (human)	Toothpaste containing SLS	oral mucosal desquamation	[ 24 ]
Mucosal reactions	Case-report	*In vivo* (human)	Toothpaste containing SLS	allergy	[ 56 ]
Mucosal reactions	triple case-report	*In vivo* (human)	Toothpaste containing SLS	inflammatory reactions of the anterior dorsal tongue	[ 26 ]
Mucosal reactions	experimental	*In vivo* (rats)	oral mucosa	Contact sensitivity-like reactions were found .in the oral mucosa	[ 27 ]
Mucosal reactions	crossover RCT	*In vivo*	The toothpastes with 1.2% SLS, 1.2% SLS + 4% betaine and only with 4% betaine were placed on buccal mucosa for 15 min	SLS: irritates the oral mucosa Betaine: does not reduce the effect of SLS	[ 28 ]
Mucosal reactions	experimental	*In vitro*	human oral mucosa cultures + SLS 0%, 0.015%, 0.15%, 0.5%, 1.0% and 1.5%	SLS can have a dual effect on the human oral epithelium	[ 29 ]
Interactions with CHX	RCT	Human	Regimen A (positive control): rinsing with CHX alone.	No significant difference in bleeding index.	[ 30 ]
			Regimen B: rinsing with CHX preceded by rinsing with an SLS-containing slurry Regimen C: rinsing with CHX preceded by tooth brushing with an SLS-containing dentifrice	Regimen B showed statistically significant higher plaque accumulation.	
Interactions with CHX	Meta-Analysis	-	4 RCTs were included:	the combined use of dentifrice and CHX mouthwash is not contraindicated.	[ 31 ]
			Comparing CHX mouthwash as a single oral hygiene intervention with the use of CHX in combination with SLS-free and with SLS-containing dentifrices	Moderate risk of bias was detected.	
Other	Experimental	*In vitro*	Adhesive (0.5% and 0.6%)+ SLS (concentration range 0.0025%-0.0075%)	The cell death was dominated by necrosis, but apoptosis was increased with SLS concentrations and was the prevailing death mechanism at SLS concentrations of 0.0075%	[ 32 ]
Other	Experimental	*In vitro*	commercially available toothpastes containing SLS	Detergents’ type in toothpastes associated with changes in *in-vitro* cell toxicity	[ 33 ]
Other	RCT	*In vivo* (human)	SLS detergents 2.0% w/v with and without 4.0% w/v betaine in distilled water in 20 volunteers, and 0.5% and 1.0% w/v SLS combined with 4.0% w/v betaine	Betaine was ineffective on the immediate mucosal impact of 0.5% and 2% SLS or 2% CAPB, but abolished the irritating effect of 1% SLS.	[ 28 ]
Other	RCT	*In vivo* (human)	The ability of Ndu tea® and Lipton® tea containing 1.2% w/v SLS	The extracts of Ndu and Lipton tea potently reduced the CFU/milliliter by SLS	[ 34 ]

RCT: Randomized clinical trial, HGFs: Human gingival fibroblasts, FTF: Fructosyltransferase, EC: Exfoliative cheilitis, W/V: Weight/Volume

CMC: critical micelle concentration, SGF: simulated gastric fluid, FeSSIF: Fed state simulated intestinal fluid, ISR: Isradipine, PVP: Polyvinyl pyrrolidone

LDAO: lauryldimethylamine N-oxide, API: Active pharmaceutical ingredient, HMPC: Hydroxy propyl methyl cellulose acetate succinate

LLPS: liquid-liquid phase separation, PSZ: Posaconazole, VPS: Vinylpolysiloxane, CAPB: Cocamidopropyl betaine, CFU: Colony Forming Unit

CHX: Chlorhexidine

## Literature Review

### Wound Healing

Oral mucosal wounds heal more rapidly and with less scar tissue formation compared to the skin wounds [ 12 ]. However, there are substances that can prolong the healing process of oral wounds. This is important specifically following the oral surgical procedures [ 13 ]. 

To study the effects of SLS on oral wound healing, Chuang *et al*. [ 14 ] demonstrated statistically significant inhibition of wound healing in an *in vitro* model. These results suggest that in the oral surgical procedures in patients consuming SLS containing dentifrices, the healing time may be prolonged [ 14 ]. However, this needs further *in vivo* investigation to be proven. Nevertheless, to be on the safe side, applying an SLS-free toothpaste is recommended in order to avoid possible negative response in early stages of healing.

### Impact on Electronic-Tongue Devices

The unpleasant taste of orally administered drugs might lead to medicine intake rejection especially in pediatric patients. Therefore, all orally used drugs have to be tested by electronic-tongues that mimic the function of the human tongue tasting [ 15 - 16 ]. Taste-masked oral liquid formulations sometimes contain substances that may harm e-tongue sensors. Based on a study by Immohr *et al*. [ 17 ], regarding the impact of SLS on oral liquids in e-tongue measurements, it has been shown that SLS changes the “test” signal sensor sets compared to the “control” sensor. However, the performance of the sensor was not damaged by this change [ 17 ].

### Effects on Free Fluoride Concentration in Oral Fluids

The aim of the application of topical fluoride is to in crease the concentration of oral free fluoride. SLS is a common component of toothpastes. Likewise, Fluoride is used in various forms in dentistry, such as mouthwashes, toothpastes, varnishes, and so on [ 18 ]. To investigate the effects of SLS on oral fluoride levels, Vogel *et al*. [ 19 ] found that SLS does not significantly affect the total saliva and plaque fluoride however, significantly increases the salivary fluid and plaque fluid fluoride by 147 and 205%, respectively. It has been suggested that manipulating non-fluoride ingredients of fluoride toothpaste and rinses, especially surfactants such as SL-S, could increase the release of fluoride from its oral re-servoirs after conventional topical fluoride therapy [ 19 ]. 

### Plaque Index

Dental plaque can be defined as an aggregation of oral bacterial species embedded in a poly-carbohydrate matrix, which is attached to the tooth surface [ 20 - 21 ]. Glycosyltransferase and fructosyltransferase are two main exo-enzymes, playing key roles in the production of extracellular polysaccharides including glucans and fructans in the presence of sucrose [ 22 - 23 ]. Extracellular polysaccharides can improve bacterial adherence and also act as a nutrient supplement in food shortage periods [ 24 - 25 ].

Steinberg *et al*. [ 26 ] in an *in vitro* study investigated the effect of various antiplaque agents including the effect of SLS on plaque accumulation. The results showed that SLS could inhibit fructan production by reducing the fructosyltransferase activity [ 26 ]. Jeong *et al*. [ 27 ], in a clinical trial evaluated the effect of three different concentrations of SLS on various plaque indices in young patients. The simplified oral hygiene index showed a reduction after 4 weeks as SLS concentration increased. The overall results supported the idea that SLS has a positive antiplaque activity [ 27 ]. However, Sälzer *et al*. [ 28 ] reported that there is an inconsiderable difference between SLS-containing and SLS-free toothpastes for controlling the plaque accumulation in the patients suffering from gingivitis. This however, seems incompatible with the previously mentioned studies.

*In vitro* animal and human studies indicated that keeping higher concentrations of fluoride surrounding the tooth might be an important factor for cariostatic protection of topical fluoride remedies [ 29 ]. In a study, it was shown that additional SLS caused no differences between the plaque mass or salivary flow rates [ 19 ]. In contrast to plaque fluid fluoride and salivary fluid, total saliva, saliva particulates, and total plaque were not significantly changed. Furthermore, the levels of total salivary fluoride were notably greater than the levels of salivary fluid fluoride for both the SLS-containing rinses and non-SLS ones [ 19 ].

### Oral Recurrent Aphthous Stomatitis

Recurrent aphthous stomatitis (RAS) is a referring mucosal condition that occurs as multiple or solitary lesions, and the most common complaint of patients is pain. Furthermore, RAS is normally resolved within 5 to 8 days [ 30 ], and recurs with an episode of three to six times in a year [ 31 ]. The etiology of RAS is unknown, but studies showed a possible relation of its occurrence with the systemic and psychological factors as well as nutrition [ 5 ]. A six-fold decrease in the life quality of the patients who suffered from RAS was reported [ 5 ]. The treatment strategy of RAS mainly relies on a good oral hygiene, which requires the consumption of oral dentifrices [ 30 ]. As mentioned before, most of the commercially available dentifrices contain SLS, thereby, its effects on RAS ulcers should be considered.

Shim *et al*. [ 5 ] compared the effects of SLS-free and SLS-containing dentifrices in subjects with RAS. They divided 90 patients into three groups and analyzed the clinical parameters (mean pain score, number of episodes, duration of ulcers, number of ulcers) after the intervention period [ 5 ]. Although there was no significant difference between the ulcer numbers and episodes, the healing duration of the ulcers and the pain score was significantly lower in the SLS-free group [ 5 ]. In another study, Alli *et al*. [ 30 ] reviewed four double-blinded RCTs as a meta-analysis. They concluded that RAS patients, who use SLS-free over SLS-containing dentifrices, might experience a reduction in the number of ulcers, duration of ulcers, number of episodes, and ulcer pain [ 30 ].

### Management of Halitosis

Halitosis is an endogenous mouth malodor identified to be related to sulphur, organic nitrogen components (amines) as well as ammonia gas [ 32 - 34 ]. Jeong *et al*. [ 35 ] in a clinical trial demonstrated the effect of detergents containing SLS on halitosis. The findings revealed that SLS could alter the gas mass as time went by. The amounts of sulfide and ammonia gasses were dropped, while in contrast to sulfide gas, the result was not significant for ammonia [ 35 ]. Similarly, Peruzzo *et al*. [ 36 ] reported a significant decrease in the amount of volatile sulphur compound, which its presence on exhaled breath causes halitosis, formation on the morning breath of the patients using SLS-containing dentifrices.

### Carrier for Various Oral Drugs (Solubilizer for Poorly Soluble Drugs)

Drug solubility and dissolution restricts its absorption [ 37 ]. It is shown that surface tension is reduced due to surfactants and following that, an improvement occurs in lipophilic drugs’ dissolution in aqueous medium [ 38 ]. Micellar solubilization with surfactants is a well-known method to improve the solubility of the poorly soluble drugs in the solid dosage forms [ 39 ]. Micelles are amphiphilic polymers with hydrophobic suitable core part and outer shell targeting the drug to the specific area [ 40 ]. For highly permeable but poorly soluble drugs like that in Bahr *et al*.’s [ 41 ] study, the optimum amount of SLS is needed for maximum drug concentrations in body fluids and better outcome.

 Alizadeh *et al*. [ 42 ] demonstrated that SLS with the formation of micelles could improve the solubility of different drugs. Among 19 drugs, they identified that acetaminophen and ketoconazole plus SLS had the least and most increasing solubility, respectively [ 42 ]. The formation of the micelles occurs above the critical micelle concentration and, most of the increase in the solubility of the drug occurs when micelles are formed. However, some drugs demonstrated improvement in solubility under critical micelle concentration [ 42 ]. Qiang *et al*. [ 2 ] also showed that spectrum SLS (Gardena, CA) improved dissolution of BILR 355 (11-ethyl-5,11-dihydro-5-methyl-8-(2-(1-oxido-4-quinolinyl) ethyl)-6H -dipyrido(3,2-b,2',3'-e) (1,4) diazepin-6-one) more effectively than Cognis (TEXAPON® K12 P PH, NF/ Ph.Eur., Düsseldorf, Germany) SLS (20% higher, 10% more dissolved drug, and less water needed). 

Chen *et al*. [ 6 ] aimed to evaluate the possible role of SLS in the bioavailability of amorphous solid dispersions. In this regard, they reported the outcomes of using SLS as a combined solubilizing agent in Posaconazole/ Hydroxy propyl methyl cellulose acetate succinate (HPMC) [ 6 ] in their experimental study; they confirmed the effect of SLS on enhanced solubility. SLS was more effective than HPMC; moreover, it could have synergism with HMPC [ 6 ]. Besides, SLS by competing with HPMC could decrease the crystallization forming of Posaconazole [ 6 ].

Shelar *et al*. [ 43 ] used SLS in combination with vitamin E tocopherol polyethylene glycol succinate to formulate a more stable nano-suspension system. Enhancement *in vitro* dissolution and *in vivo* pharmacokinetic profile occurred compared to pure isradipine suspension. Hence, the isradipine nano-suspension confirmed to be a promising formulation method for the increase of isradipine oral bioavailability. This study showed that particle size reduction can change isradipine absorption in the gastrointestinal tract therefore; nano-suspension technology is responsible for boosting oral bioavailability in rats [ 43 ].

In the study of Shidhaye *et al*. [ 44 ] among different penetration enhancers, formulations including 1% SLS showed a good penetration of pravastatin sodium through the mucosa. In addition, the histopathological evaluation did not display any buccal mucosal damage like necrosis [ 44 ]. In another study, Ates *et al*. [ 45 ] considered using SLS as a means to modulate cellular tight junctions of intestinal epithelial cells as it is proven to open cellular tight junctions reversibly. This action was done as an effort to enhance the permeability of acyclovir; an antiviral drug with little absorption from the gastrointestinal pathway, through intestinal epithelial membrane permeation-enhancing effect of SLS was notable only in the ileum [ 40 ].

SLS has also been used in the process of making novel sustained-release drugs [ 51 ]. Hydrophilic drugs are absorbed rapidly in the gastrointestinal tract and their absorption is dependent on the pH of the medium. As a result, if not used carefully, these drugs seem to get to toxic levels in a short period [ 51 ]. In a study, Kasashima *et al*. [ 46 ] used Mirabegron, a drug primarily used for treating over-reactive bladder in a phosphate buffer (pH=6.8). Among the other substances used in the study, SLS was the most suitable substance in terms of oral sustained-release [ 46 ]. This formulation and its *in vivo* absorption and bioavailability were compared to that of Mirabegron solution in Beagle dogs, which were studied in another research also conducted by Kasashima *et al*. [ 47 ]. Mirabegron solution might reach toxic levels rapidly, while lauryl sulfate (LS) salt/complex suspension and microparticle LS salt/complex suspension do not have distinctive peaks in plasma concentration of the drug while used orally [ 47 ]. LS salt/complex suspension showed differences in maximum plasma concentration among fasting and fed dogs. This effect can be eliminated by using microparticle LS salt/ complex suspensions [ 47 ].

### Periodontal Treatment

Periodontitis is a prevalent oral infection causing irreversible destruction of tooth-supporting structures [ 48 ]. Periodontal disease is initiated by localized inflammation of gums (gingivitis), which is etiologically linked to dental plaque. It seems that SLS is capable of making gingiva and mucosa vulnerable to exogenous antigens by denaturing proteins of mucin [ 49 ].

According to an *in vitro* study designed by Okte and Bal [ 50 ], applying SLS to cementum surface can lead to its physical change. 5-minute exposure resulted in exposing collagen and dentinal tubules. It was concluded that further studies are needed to evaluate SLS effects on the regeneration ability of tooth-supporting tissues in teeth with periodontal disease [ 50 ]. In another study on the patients with moderate gingivitis, SLS-free and SLS-containing toothpastes showed approximately the same efficacy on gingival health scores and gingival abrasion. Having mentioned that, only SLS-containing one led to increased taste satisfaction among the patients. As a result, non-SLS dentifrices might be an acceptable alternative for SLS-containing ones in patients diagnosed with gingivitis [ 28 ].

### Exfoliative Cheilitis

Exfoliative cheilitis (EC) is a scarce disease that affects the vermilion of one or both lips by continuous production and therefore, desquamation of thick keratin scales [ 51 ]. EC's onset seems to be associated with different elements such as stress, psychological status, personality disorders, and so on. Nevertheless, the main etiology is still unknown [ 51 - 52 ]. Thongprasom [ 53 ] reported a 19-year-old female case with EC. A patch test revealed that the patient was allergic to SLS. Slow healing occurred after cessation of SLS-containing toothpaste and applying glycerin borax and hydrogen peroxide (1%) mouthwash [ 53 ]. Similar reports also pointed out the occurrence of EC in reaction to toothpastes [ 54 - 55 ]. However, these studies have not proven SLS to be the main cause of the condition. Therefore, the literature is still inconclusive regarding the hypothesis of SLS being a risk factor of EC. 

### Mucosal Reactions

One of the conditions that can cause erosive and ulcerative lesions in the oral cavity is hypersensitivity reaction to substances [ 56 ]. SLS is known to be an anionic surfactant involved in the destruction of the oral mucosal epithelium and has the ability to cause contact sensitivity-like reactions, as well as allergic contact reactions and irritating reactions on oral mucosa [ 10 , 57 - 59 ]. Neppelberg *et al*. [ 56 ] showed that SLS could have a dual effect on the human oral epithelium. According to the results obtained; at low doses of SLS (<0.015%), epithelial cell proliferation occurs and the epithelial thickness increases, while high doses of SLS (≥0.015%) lead to epithelial cell degradation [ 56 ]. Allergies to toothpastes containing SLS have also been shown to cause oral lesions [ 57 ]. Even in people with no history of allergic reactions to SLS, some specific SLS compounds develop nonspecific erythematous irritating reactions [ 57 ]. It has been shown that the use of toothpaste containing SLS causes more mouth ulcers in patients than the use of toothpastes without it [ 60 ]. Inflammation from the products such as toothpastes containing SLS can cause leukoedema with mucosal deposition. Oral epithelial de-scaling is more likely to occur when the SLS concentration in the product is higher [ 10 ]. 

Oral mucosa responds to lower doses of SLS compared to skin [ 8 ]. Mucosal and skin permeability are increased by SLS, and triclosan has the ability to suppress the immune system [ 8 ]. Triclosan (2,4,4'-trichloro-2'-hydroxyl-diphenyl ether) is a lipid-soluble substance with antibacterial activity used in cosmetics and soaps. Mustafa *et al*. [ 61 ] showed that triclosan, in addition to its antibacterial capacity, also has anti-inflammatory effects. Furthermore, it has a protective effect against the reactions caused by SLS [ 61 ]. Although there are evidences of allergic and toxic reactions caused by systemic intake of SLS, there is no scientific finding supporting that SLS is a carcinogen and it is not listed as a carcinogen by the International Agency for Research on Cancer [ 58 , 62 ].

### Effects on Enamel Erosion

Dental erosion is a process that is influenced by many factors and identified by the chemical demineralization of enamel, created by acids, and chelating factors [ 63 ]. Before its contact with the enamel, the acid must be released through the pellicle [ 29 ]. Enamel pellicle is a free bacterial film that coats dental structures and is composed of many proteins such as glycoproteins, mucins, and proline-rich proteins [ 64 - 65 ]. In addition, it acts as a barrier that prevents contact between the tooth surface and acids [ 63 ]. Therefore, it protects enamel against demineralization [ 64 , 66 ]. SLS can affect the availability of fluoride ions and their binding to dental structures. This suggests that SLS competes with fluoride ions for calcium-binding areas, preventing or reducing the amount of sodium fluoride (NaF), thus decreasing its protective effect. Furthermore, SLS reduces the NaF discharge on enamel and augments the solubility of the calcium fluoride (CaF2) precipitated pattern [ 67 ] which is the main part responsible for NaF protection from erosion [ 68 - 69 ]. According to the study by Zanatta *et al*. [ 70 ] regarding the effect of fluoride and surfactants such as SLS on enamel erosion, SLS reduced the protection of fluoride in the initial erosion, but this destructive effect did not last while maintaining the erosive cycle. Therefore, SLS does not seem to threaten the protection provided by the fluoride and the pellicle in long-lasting erosive conditions.

### Cytotoxicity

Mouthwashes and toothpastes are generally used as plaque control adjuncts, which may contain toxic ingredients for oral tissues [ 71 ]. One of the detergents in the composition of toothpastes is SLS and it has been shown to have a significant toxic results *in vitro* [ 72 ]. It can change the proteins of oral mucosal tissues [ 73 ] and increases the blood circulation of the gingiva [ 74 ]. 

Based on the study of Cvikl *et al*. [ 72 ] in which the effects of toothpaste components on cell viability were examined, the toothpaste containing SLS completely compromised cell viability. Moreover, in the study of Tabatabaei *et al*. [ 75 ], SLS showed to be the highest toxic ingredient among the other toothpaste ingredients and it presented more than 90% toxicity at whole concentrations on human gingival fibroblasts. 

Piret *et al*. [ 76 ] suggested that gel formulation containing the 2% W/W of SLS, could be considered safe for the skin, eyes, buccal mucosa, rectum, male, and female genital organ. Therefore, it was proposed that this gel formulation could be a potential choice to use as an antimicrobial means against sexually transmitted pathogens such as HIV-1 [ 76 ].

### Effects on Saliva

Xerostomia or dry mouth is defined as an uncomfortable feeling of dryness in the oral cavity [ 77 ]. Dry mouth can be caused by diminished salivary function although most patients do not manifest any objective signs of hypo-function [ 78 ]. Different oral health products such as dentifrices, mouth rinses, and gels can take a part as saliva stimulators or alternatives [ 79 ]. Hwa-Yeong Jeong *et al*. [ 27 ] found a negative association between SLS concentration and salivary flow in their clinical trial in young patients. However, no correlation was found between the salivary viscosity and pH [ 27 ]. It is reported that the patients with dry mouth are more satisfied with using both SLS- betaine-containing dentifrices [ 80 ]. Rantanen *et al*. [ 59 ] measured the mucosal irritation of SLS-containing dentifrices with/without betaine by visual and electrical methods. Both experimental dentifrices showed irritating effects and no obvious difference was found when betaine was in combination with SLS [ 59 ]. In contrast with the previous studies, Rantanen *et al*. [ 9 ] also conducted another randomized clinical trial and reported that betaine-containing dentifrices could aid with dry lips as an example of xerostomia symptoms. All dentifrices including SLS-containi-ng ones showed no side effects such as mucosal irritation during their study [ 9 ]. It was concluded that irritation effects on oral mucosa shown in previous studies might be because of other ingredients in toothpastes or a result of their synergetic or additive effects on SLS [ 59 ].

### Interaction with Chlorhexidine Mouthwashes

The possible interaction between SLS-containing toothpastes and chlorhexidine (CHX) mouthwashes is discussed in the literature [ 81 - 84 ]. This was raised by an *in vivo* classic study in 1989, where it is suggested that due to interaction between SLS and CHX, a 30-minute window should be defined between tooth brushing and CHX use [ 83 ]. This was later supported by a systematic review, in which the authors suggested an interval of 30 minutes to 2 hours [ 84 ]. However, a more recently published meta-analysis concluded that there is no significant reduction in the efficacy of CHX mouthwash following tooth brushing, if properly rinsed with water after brushing [ 82 ]. This seems to be supported by the results of a randomized triple-arm study where authors reported no significant reduction in the anti-plaque efficacy of CHX (0.2%) rinse preceded by SLS-containing toothpaste if rinsing is performed with a non-SLS containing liquid (ideally water) [ 81 ].

### Other Impacts on Oral Cavity

Detergents such as SLS, play a role in foaming and dissolution of the components in toothpastes [ 72 - 73 , 85 ]. Previous studies have shown that this substance interrupts the integrity of the cell membrane [ 86 ]. It is suggested that SLS affects the membrane due to its amorphous solid dispersion property and therefore, can have antimicrobial properties and on the other hand, is a danger to the safety of toothpastes [ 6 , 72 ].

In a study by Charles O *et al*. [ 87 ], the antimicrobial properties of SLS were investigated. In their study, the role of this substance as a supplement to tea extract was assessed by comparing the extract of 2 commercially available teas and the SLS added tea [ 87 ]. Finally, it was found that the tea extracts are able to reduce bacterial colony formation, and SLS has a synergic impact on this regard increasing the antimicrobial effect of teas [ 87 ]. 

Rantanen *et al*. [ 59 ] in a double-blinded clinical trial examined the role of betaine in SLS-containing dentifrices. They used electrical impedance spectrum in terms of four indices that indicate mucosal irritation including impedance magnitude index (MIX), impudence’s phase index (PIX), imaginary part index (IMIX), and real part of impedance index (RIX) to emphasize different aspects of the impedance properties of the human oral mucosa. SLS at 0.5% and 1% concentrations increased irradiation indices including MIX, PIX, and IMIX, but at 2% concentrations increased all indices [ 59 ]. The results of this study also showed that SLS irritation increased over time. The concomitant effect of betaine at 1% SLS concentration reduced irritation indices and had no significant effect at 0.5% and 2% concentrations [ 59 ].

SLS can alter the properties of human oral mucosal cell walls and therefore, can affect cell viability. In a study by Moore *et al*. [ 88 ], incubation of keratinocytes with SLS in 2 minutes reduced viability. The concentration of cytotoxic IC50, which demonstrates how much drug is required to inhibit a biological process by half [ 89 ] was 0.002% for corneal epithelial cells, 0.005% in submandibular salivary acinar cells and 0.0014% in keratinocytes *in vitro* [ 90 - 92 ]. In an animal study by Roll *et al*. [ 91 ], cytotoxicity was shown to be one of the complications observed in SLS-exposed cells. Cytotoxicity occurs in cells throughout two mechanisms including apoptosis and necrosis [ 91 ]. In their study, the preponderance of the cytotoxic effect of SLS was due to necrosis and conversely, apoptosis had a less prominent role in this phenomenon. Irradiation results showed that the rate of cell death in cells was dose-dependent on SLS [ 91 ]. In addition, Cvikl *et al*. [ 72 ] noted that toothpaste, containing SLS, was more cytotoxic to fibroblasts and epithelial cells than other compounds such as Cocamidopropyl betaine (CAPB) and strearch-20 that are detergents similar to SLS and used in some toothpastes. The cytotoxicity of SLS based on the above mentioned *in vitro* studies, cannot be generalized to its *in vivo* impacts, as it has a protective effect on saliva and the immune system. Hence, more studies are needed to clarify this argument.

## Discussion

When it comes to exploring the impact of SLS on periodontal and oral health, the dentifrices and mouthwashes containing this substance are the main topics to address. This review study was conducted to identify and summarize both the positive and adverse effects of SLS that are reported in the dental-related literature.

Our search strategy and review verified that regarding the influence of SLS on oral mucosa and epithelium, a wide range of drugs are reported to have an increased bioavailability when combined with SLS [ 2 , 39 - 40 , 43 , 45 , 65 ] such as posaconazole, vitamin E tocopherol, polyethylene glycol succinate, pravastatin sodium, acyclovir, and insulin. Likewise, several studies have shown the positive outcomes of SLS-containing products on periodontal patients [ 19 , 48 - 49 ]. These evidences also are strengthened by the fact that SLS can improve the control of plaque accumulation [ 71 ]. Additionally, positive impacts on the reduction of halitosis [ 32 , 35 - 36 ], elimination of oral bacteria and increased free fluoride levels has been reported. However, the level of evidence supporting each of aforementioned positive outcomes is weak and high quality human-studies are extremely scarce. Nevertheless, this paper might provide a framework for further studies and the gaps that should be filled in this regard.

To discuss the negative impacts of SLS, the consumption of SLS can increase the duration of wound healing process [ 12 - 14 ]. It is reported that it decelerates the healing process of conditions like EC; however, concerning the absence of sufficient information, further studies are required [ 53 - 55 ]. Similarly, this surfactant can also affect the oral epithelium negatively, resulting in allergic and hypersensitivity reactions in some patients [ 8 , 93 ]. Some of the examples are erythematous irritating reactions, mouth ulcers, oral inflammation, and leukoedema following consumption of SLS-containing toothpastes and products. In terms of effects of this surfactant on enamel erosion, there are controversial evidences, which require further human studies [ 64 - 67 , 70 ]. Likewise, concerning the cytotoxicity of SLS, studies can be divided into two groups; the first group suggests that it is a safe component [ 64 - 65 ] while other studies consider it as a cytotoxic agent [ 67 , 70 ]. In addition, SLS-containing dentifrices caused more pain and discomfort in patients who suffer RAS [ 30 ]. Therefore, prescription of SLS-free agents for these patients is highly suggested. Overall, the proven drawbacks of this substance consist of aggregation in RAS patients; reduced oral wound healing capability, and oral mucosal irritation, which based on the current evidence are applicable to human subjects [ 5 , 30 ]. Thus, the other abovementioned negative aspects require higher level of evidence and human studies. 

When studying the impact of chemical agents on oral tissue, a crucial aspect is to determine the clearance of the agent from the oral tissue. Since most of the exposure of oral tissue to SLS results from the consumption of dentifrices, the half-life and clearance of SLS should be considered. Fakhry-Smith *et al*. [ 94 ], by using high performance liquid chromatography, reported that 86% of the amount of SLS is recovered from the oral cavity after tooth brushing within the first 10 minutes. This is inconsistent with the results of the studies that propose a 30-minute to 2-hour window between SLS and CHX use [ 81 ]. Thus, it seems that based on these findings proper rinsing of the mouth with water following SLS exposure will prevent possible detrimental outcomes.

Lastly, it should be reminded that concerning the narrative review framework of this study, the suggested results should be applied in practice cautiously, while ideally, further systematic reviews and meta-analyses can serve superior outcomes. Nonetheless, since the included studies in this paper were mostly animal and *in vitro* studies, there are not sufficient evidences considering the impact of SLS on human periodontal and oral health. Therefore, except the very few topics, further human studies are highly recommended. 

## Conclusion

Within the limitations of this review study, SLS can serve positive outcomes in terms of increasing bioavailability of medications, plaque control, and halitosis. However, the exacerbation of RAS condition, compromising oral wound healing, and irritation of the oral mucosa are concerned among the adverse effects. Moreover, there is a lack of sufficient controlled trials and human studies on the preponderance of these possible impacts.

## Conflict of Interest

The authors declare that they have no conflict of interest.
